# Disease-linked connexin26 S17F promotes volar skin abnormalities and mild wound healing defects in mice

**DOI:** 10.1038/cddis.2017.234

**Published:** 2017-06-01

**Authors:** Eric Press, Katanya C Alaga, Kevin Barr, Qing Shao, Felicitas Bosen, Klaus Willecke, Dale W Laird

**Affiliations:** 1 Department of Physiology and Pharmacology, University of Western Ontario, London, ON, Canada; 2 Department of Anatomy and Cell Biology, University of Western Ontario, London, ON, Canada; 3 LIMES (Life and Medical Sciences Institute), Molecular Genetics, University of Bonn, Bonn, Germany

## Abstract

Several mutant mice have been generated to model connexin (Cx)-linked skin diseases; however, the role of connexins in skin maintenance and during wound healing remains to be fully elucidated. Here we generated a novel, viable, and fertile mouse (Cx26^CK14-S17F/+^) with the keratitis-ichthyosis-deafness mutant (Cx26S17F) driven by the cytokeratin 14 promoter. This mutant mouse mirrors several Cx26-linked human skin pathologies suggesting that the etiology of Cx26-linked skin disease indeed stems from epidermal expression of the Cx26 mutant. Cx26^CK14-S17F/+^ foot pad epidermis formed severe palmoplantar keratoderma, which expressed elevated levels of Cx26 and filaggrin. Primary keratinocytes isolated from Cx26^CK14-S17F/+^ neonates exhibited reduced gap junctional intercellular communication and migration. Furthermore, Cx26^CK14-S17F/+^ mouse skin wound closure was normal but repaired epidermis appeared hyperplastic with elevated expression of cytokeratin 6. Taken together, we suggest that the Cx26S17F mutant disturbs keratinocyte differentiation and epidermal remodeling following wound closure. We further posit that Cx26 contributes to epidermal homeostasis by regulating keratinocyte differentiation, and that mice harboring a disease-linked Cx26 mutant display epidermal abnormalities yet retain most wound healing properties.

Gap junction channels allow for direct intercellular communication by facilitating the passage of small molecular messengers between the cytosol of adjacent cells.^1^ Individual cells may also exchange signaling molecules with the extracellular environment through hemichannels at the cell membrane.^2^ Gap junction channels are composed of docked hemichannels from adjacent cells, each of which contains six oligomerized connexin (Cx) subunits. Connexins comprise a family of transmembrane proteins that have large clinical significance due to the discovery of many disease-causing mutations within connexin encoding genes. Mutations in the genes encoding Cx26, Cx30, Cx30.3, Cx31, and Cx43 are extensively linked to a variety of hereditary skin diseases.^3, 4^ Notably, mutations in the genes encoding Cx26 and Cx30 cause syndromic diseases where patients suffer from hearing loss and a broad range of skin abnormalities that vary in localization and severity.^3, 5, 6^ Interestingly, Cx26 mutants with similar functional characteristics often lead to comparable phenotypes, suggesting specific functional anomalies are closely tied to disease outcomes.^5, 7^ Not surprisingly, Cx26 is expressed in the cochlea and epidermis, and shares a similar localization pattern with Cx30 in the epidermal strata,^8^ and likely form heteromeric channels between keratinocytes.

The human epidermis expresses up to 7 distinct connexin proteins in overlapping populations of keratinocytes highlighting the complexity of gap junctional intercellular communication (GJIC) in this tissue.^9^ Genetically modified mouse models previously demonstrated that persistent epidermal Cx26 expression promoted a hyperproliferative, inflammatory response suggesting a delicate balance of individual connexins may be required for epidermal health. A key factor likely involved in regulating keratinocyte differentiation is an increasing intracellular Ca^2+^ gradient between keratinocytes of the stratum basale and stratum granulosum.^10, 11^ Gap junction channels are known to pass signaling molecules such as IP_3_ that regulate intracellular Ca^2+^ handling, which may have a profound influence on the synthesis and assembly of skin barrier components such as filaggrin and ceramides.^12, 13^ Furthermore, it is well known that following cutaneous wounding, multiple connexins including Cx26, Cx30, and Cx43, are dynamically regulated within the epidermis suggesting they have a major role in coordinating wound healing events such as keratinocyte proliferation, migration, and differentiation.^14, 15^ In fact, numerous studies employing Cx43 knockdown strategies have demonstrated beneficial wound healing outcomes,^16, 17, 18, 19, 20^ which strongly promotes Cx43 as a therapeutic target for chronic wounds. Despite the evidence supporting the influence of connexins on epidermal health, the role of Cx26 in the skin is not completely understood, particularly in the context of healing wounds.

Several Cx26 mutants, including the S17F mutant, are linked to keratitis-ichthyosis-deafness syndrome (KIDS); a rare and severe autosomal dominant disease featuring generalized dry/scaly skin, patchy erythematous keratoderma and palmoplantar keratoderma (PPK).^21^ In some cases, frequent cutaneous infections lead to fatal septicemia early in life.^21^ *In vitro* ectopic expression studies have revealed hyperactive or ‘leaky’ hemichannels as a likely pathogenic characteristic of KIDS mutants.^22, 23^ Interestingly, the S17F mutant does not form functional gap junction channels or hemichannels on its own,^22, 24^ but rather forms heteromeric hyperactive hemichannels when co-expressed with wild-type Cx26 or Cx43 (ref. 22). To understand how these molecular traits translate *in vivo*, a mutant mouse that globally expresses the S17F mutant replicates several KIDS phenotypes in addition to features typically associated with separate Cx26-linked diseases.^25^ However, because these mice have low viability and it is still unknown how the mutant specifically affects the epidermis, we generated a tissue-specific mouse that harbors the S17F mutant in the epidermis (Cx26^CK14-S17F/+^). Although numerous abnormal cellular characteristics of Cx26 mutants have been identified, and several mutant mice have been generated to model human connexin-linked skin disorders, the etiology of these diseases as well and their impact on cutaneous wound healing remains poorly understood.

Therefore, by using our novel Cx26^CK14-S17F/+^ mouse (hereafter noted as S17F/+), we aimed to assess how Cx26S17F produces skin disease, and to gain further insight into the role of Cx26 in healthy skin. We found that Cx26S17F, produced severe PPK, wherein expression of Cx26 as well as filaggrin was elevated in foot pad skin indicating deregulated differentiation. Moreover, primary keratinocytes from Cx26^CK14-S17F/+^ mice had reduced gap junctional coupling and reduced migration. Lastly, Cx26 mutant mice displayed abnormal epidermal remodeling following wound closure but retained most of the properties associated with active wound healing.

## Results

### Epidermal expression of Cx26S17F results in severe skin features plus comorbidities

**Figure 1:**
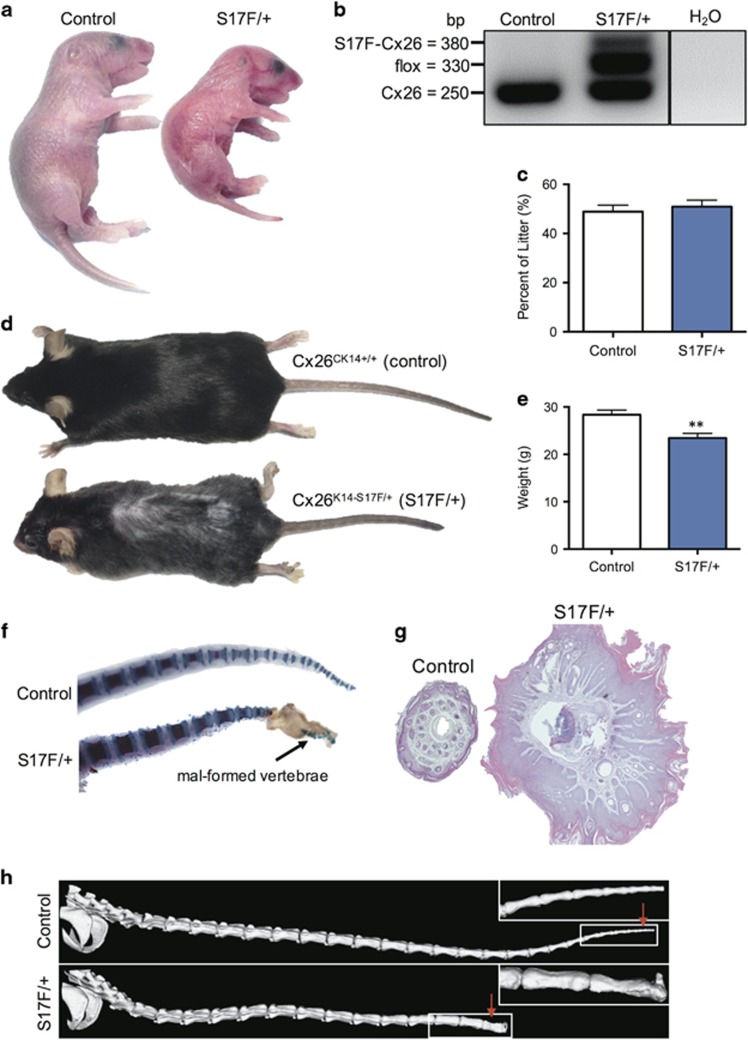
S17F/+ mice mimic KIDS skin characteristics and have several additional phenotypes. (**a**) S17F/+ neonates were smaller and visually distinguishable from control littermates. (**b**) Skin sample PCR amplification confirms the heterozygous expression of Cx26S17F in S17F/+ mice. (**c**) Litters contained equal portions of S17F/+ and control pups with no loss of mouse viability (unpaired *t*-test, Control: *n*=157, S17F/+: *n*=148). (**d**) Representative photos of 3-month-old S17F/+ and control mice reveal moderate differences in size but a pronounced tail phenotype. (**e**) Whole mouse weights at 3 months of age showed S17F/+ mice were ~15% smaller (unpaired *t*-test, **P<0.01, *N*=17). (**f**) Skeletal stains of P7 tails revealed vertebral malformations underlying the tail phenotype in S17F/+ mice. (**g**) Cross-section of a distal tail paraffin section stained with hematoxylin and eosin revealed grossly thickened epidermis. (**h**) *μ*CT scans of 3-month-old mouse tails revealed vertebral abnormalities in S17F/+ mice. Red arrows in (**h**) denote the relative locations of cross sections in (**g**)

S17F/+ mice harboring the mutated *Gjb2* gene in cells expressing cytokeratin 14 (basal keratinocytes) resulted in a conditional mutant mouse that expressed the S17F mutant under the control of the endogenous Cx26 promoter in cells of the keratinocyte lineage. This expression profile was capable of reproducing KIDS skin features in a novel mutant mouse in addition to several intriguing phenotypes (Figure 1). S17F/+ pups were visibly smaller and had red, dry, scaly skin (Figure 1a). PCR analysis of skin tissue confirmed the heterozygous expression of Cx26S17F (Figure 1b). Contrary to global Cx26^S17F/+^ mice,^25^ our tissue-defined S17F/+ mice had no loss of viability and were born in equal proportion to wild-type littermates (Control) (Figure 1c). Interestingly, S17F/+ mice were approximately 15% smaller by 3 months of age (Figure 1e), and had a shortened tail (Figure 1d) which coincided with the formation of a cutaneous bulb at the distal tail by post-natal day 7 (Figures 1f and g). This appeared to impair proper vertebral formation within the bulb preventing normal tail growth (Figure 1h).

### Cx26^CK14-S17F/+^ neonates display a functional epidermal barrier

**Figure 2:**
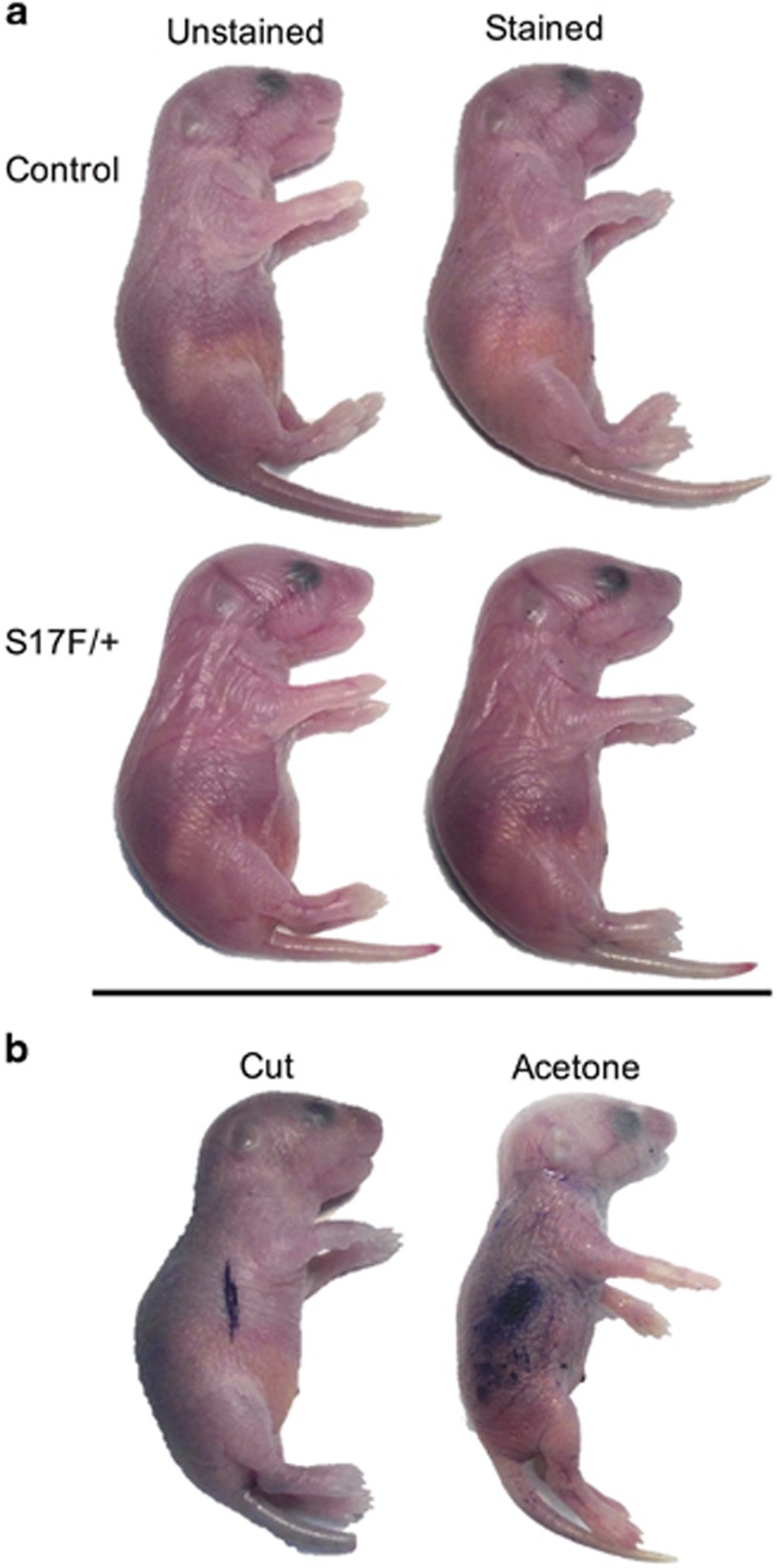
S17F/+ neonates display an intact epidermal barrier. P2 neonates were submerged in an aqueous toluidine blue solution to assess epidermal barrier permeability. Similar to control littermates, S17F/+ neonates displayed no epidermal staining indicating a functional barrier (**a**). To demonstrate epidermal staining from a defective barrier, neonatal epidermis was cut or treated with acetone (**b**). *N*=6

To test the epidermal barrier of the S17F/+ mice, we submerged euthanized neonates in an aqueous toluidine blue solution and observed epidermal staining. We found that similar to littermate controls, S17F/+ epidermis did not stain blue indicating little to no epidermal penetration of the water-soluble dye (Figure 2). However, epidermal staining was evident in areas of the skin that had been cut, or treated with acetone (Figure 2) which disrupts lipids in the epidermal barrier.^26^ This suggests that neonatal S17F/+ epidermis indeed forms an effective water barrier.

### Cx26S17F disrupts keratinocyte differentiation in foot pad epidermis

**Figure 3:**
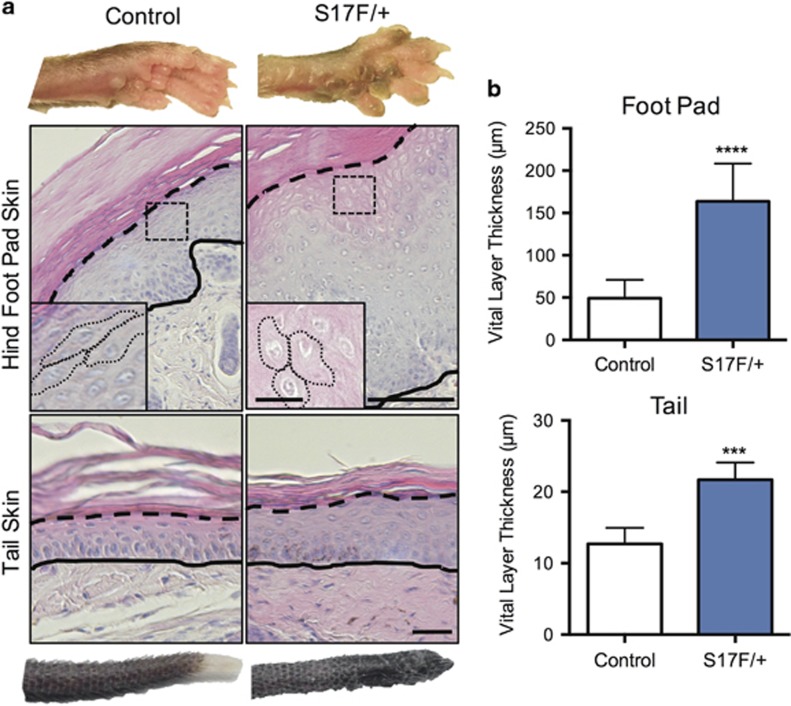
S17F/+ mice display thicker foot pad and tail epidermis. (**a**) 3-month-old S17F/+ mice exhibit severe foot pad epidermal thickening including abnormal non-squamous keratinocytes in suprabasal layers (insets) as well as thicker tail epidermis compared to controls (**b**) (unpaired *t*-test, ***P<0.001, ****P<0.0001, *N*=7). Complete and dashed lines denote the dermis-epidermis boundary and stratum granulosum-corneum boundary, respectively. Scale bar in (**a**) (upper), 50 *μ*m (inset=10 *μ*m); (**a**) (lower), 10 *μ*m

Severe PPK was clearly visible in adult S17F/+ mice in which foot pad epidermis displayed protruding hyperpigmented calluses (Figure 3a). Histological examination confirmed a gross thickening of vital epidermal layers (stratum basale, spinosum, and granulosum) (Figures 3a and b). In addition to the bulbous region at the distal tail, middle regions of tail epidermis also displayed moderate thickening of these vital layers (Figure 3b).

**Figure 4:**
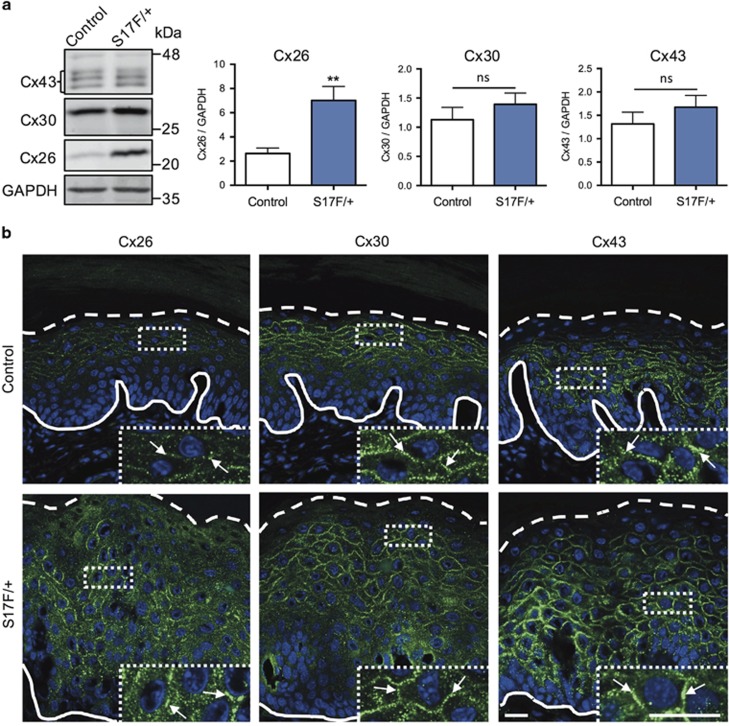
S17F/+ foot pad skin exhibits deregulated connexin expression. (**a**) Foot pad skin lysates from 3-month-old S17F/+ mice exhibited elevated levels of Cx26 expression compared to controls (unpaired *t*-test, **P<0.01, ns=*P*>0.05, *N*=10). (**b**) Cryosections of 3-month-old S17F/+ foot pad epidermis revealed that Cx26, Cx30, and Cx43 formed abundant gap junctions in a broad range of keratinocyte layers. Complete and dashed lines denote the dermis-epidermis boundary and stratum granulosum-corneum boundary, respectively. Scale bar, 20 *μ*m (inset=20 *μ*m)

**Figure 5:**
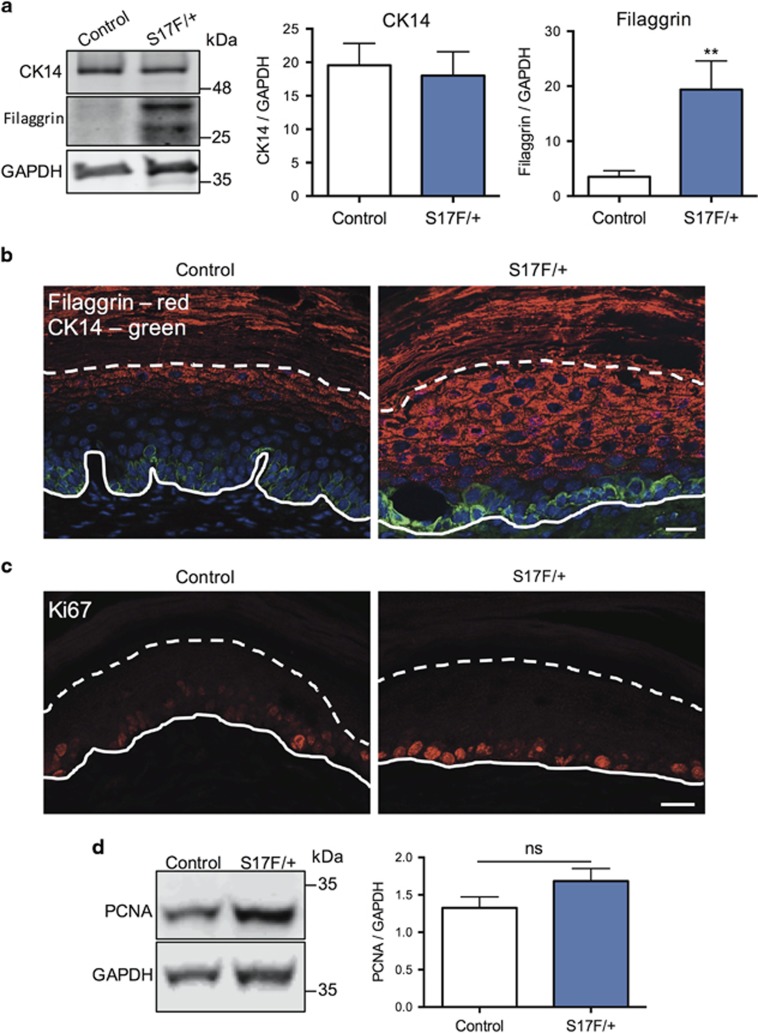
S17F/+ mice display abnormal keratinocyte differentiation in foot pad epidermis. (**a**) Foot pad skin lysates from 3-month-old S17F/+ mice have elevated filaggrin, but normal keratin 14 levels compared with controls (unpaired *t*-test, **P<0.01, *N*=10). (**b**) 3-month-old foot pad epidermis labeled for filaggrin (red) and cytokeratin 14 (green) revealed elevated filaggrin labeling of suprabasal keratinocytes in S17F/+ epidermis. (**c**) 3-month-old foot pad epidermis labeled for Ki67 (red) and lysates immunoblotted for PCNA (**d**) demonstrated unaltered levels of cell proliferation in S17F/+ epidermis (unpaired *t*-test, ns=*P*>0.05, *N*=10). Complete and dashed lines denote the dermis-epidermis boundary and stratum granulosum-corneum boundary, respectively. Scale bar in (**b** and **c**)=20 *μ*m

Tissue lysates of hind foot pad skin from 3-month-old S17F/+ mice showed elevated levels of Cx26 (Figure 4a). While no differences in Cx30 or Cx43 levels were detected in mutant epidermis, all three connexins had large overlapping distribution profiles that were clearly distinguishable from control epidermis (Figure 4b). Each connexin formed abundant gap junction plaques indicating that the Cx26S17F mutant does not impair connexin trafficking *in vivo.* S17F/+ epidermis also had normal levels of cytokeratin 14 (expressed in basal keratinocytes) but a greater amount of filaggrin (expressed in the stratum granulosum and corneum) which was found in numerous suprabasal keratinocyte layers (Figures 5a and b). Epidermal proliferation was not affected based on Ki67 (Figure 5c) and proliferating cell nuclear antigen (PCNA) assessment (Figure 5d). Together these findings suggest epidermal Cx26S17F disrupts keratinocyte differentiation and promotes the formation of PPK in mice.

### Primary keratinocytes harvested from S17F/+ mice exhibit reduced GJIC and collective cell migration

**Figure 6:**
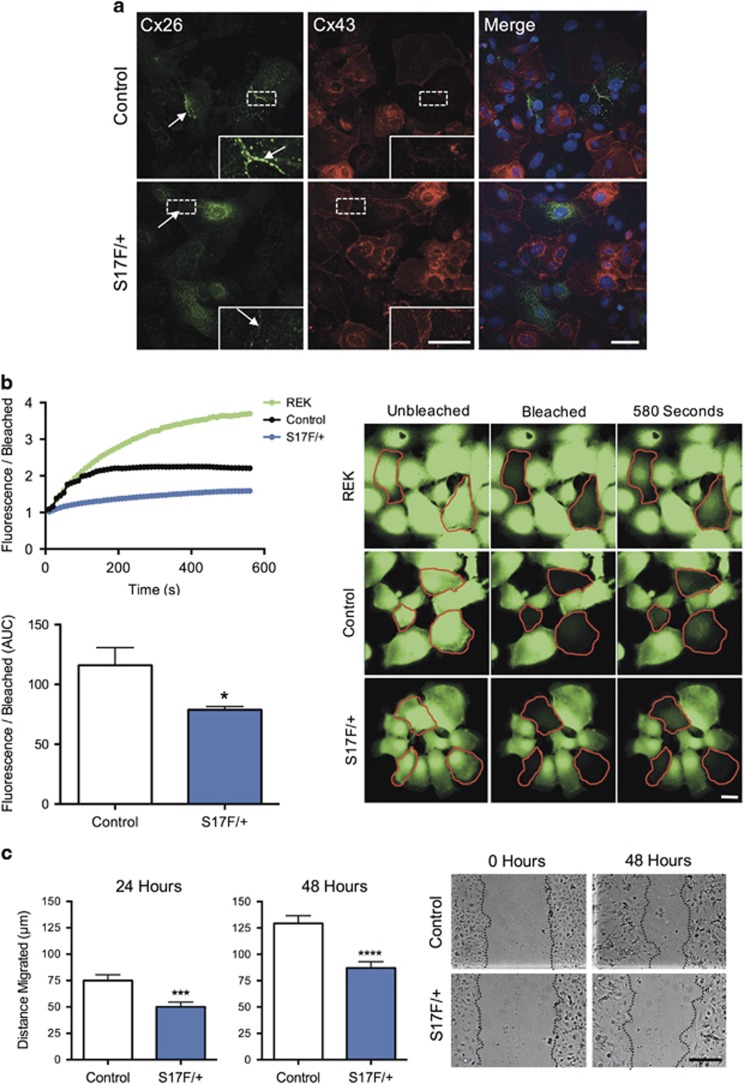
Keratinocytes isolated from S17F/+ neonates have reduced GJIC and collective cell migration. (**a**) S17F/+ keratinocyte cultures appeared to form fewer and smaller Cx26 gap junctions between cells. (**b**) S17F/+ keratinocytes have reduced calcein-AM fluorescence recovery after photobleaching compared to controls indicative of reduced GJIC (photobleached cells are outlined in red) (unpaired *t*-test, *P<0.05, *N*=3). (**c**) Collective keratinocyte migration in response to scratch wounds was reduced in S17F/+ cultures compared to controls (unpaired *t*-test, ***P<0.001, ****P<0.0001, *N*=4). Scale bar in (**a**)=20 *μ*m (inset=10 *μ*m), (**b**)=10 *μ*m, (**c**)=100 *μ*m

Keratinocytes were harvested from newborn mice to assess the influence of physiological regulation of Cx26S17F on keratinocytes. Similar to a well-characterized rat epidermal keratinocyte (REK) cell line (Supplementary Figure 1A), nearly all primary mouse keratinocytes labeled thoroughly with cytokeratin 14 and intermediate filaments were clearly visible under high magnification (Supplementary Figure 1B), indicating a highly pure keratinocyte population. In dense clustered regions, cells stratified and increased filaggrin expression, mimicking the behavior of stratified keratinocytes in live epidermis (Supplementary Figure 1B). Keratinocyte cultures were incubated in media containing 1.4 mM CaCl_2_ for 24 h to stimulate differentiation and the subsequent expression of Cx26. Compared with controls, S17F/+ cultures appeared to form fewer and smaller Cx26 gap junction plaques between cells, and demonstrated reduced fluorescence recovery following photobleaching (Figures 6a and b). Furthermore, S17F/+ cultures displayed reduced collective migration in response to scratch wounds (Figure 6c). Together these findings suggest Cx26 intercellular communication may influence keratinocyte migration.

### S17F/+ mice retain most cutaneous wound healing properties

**Figure 7:**
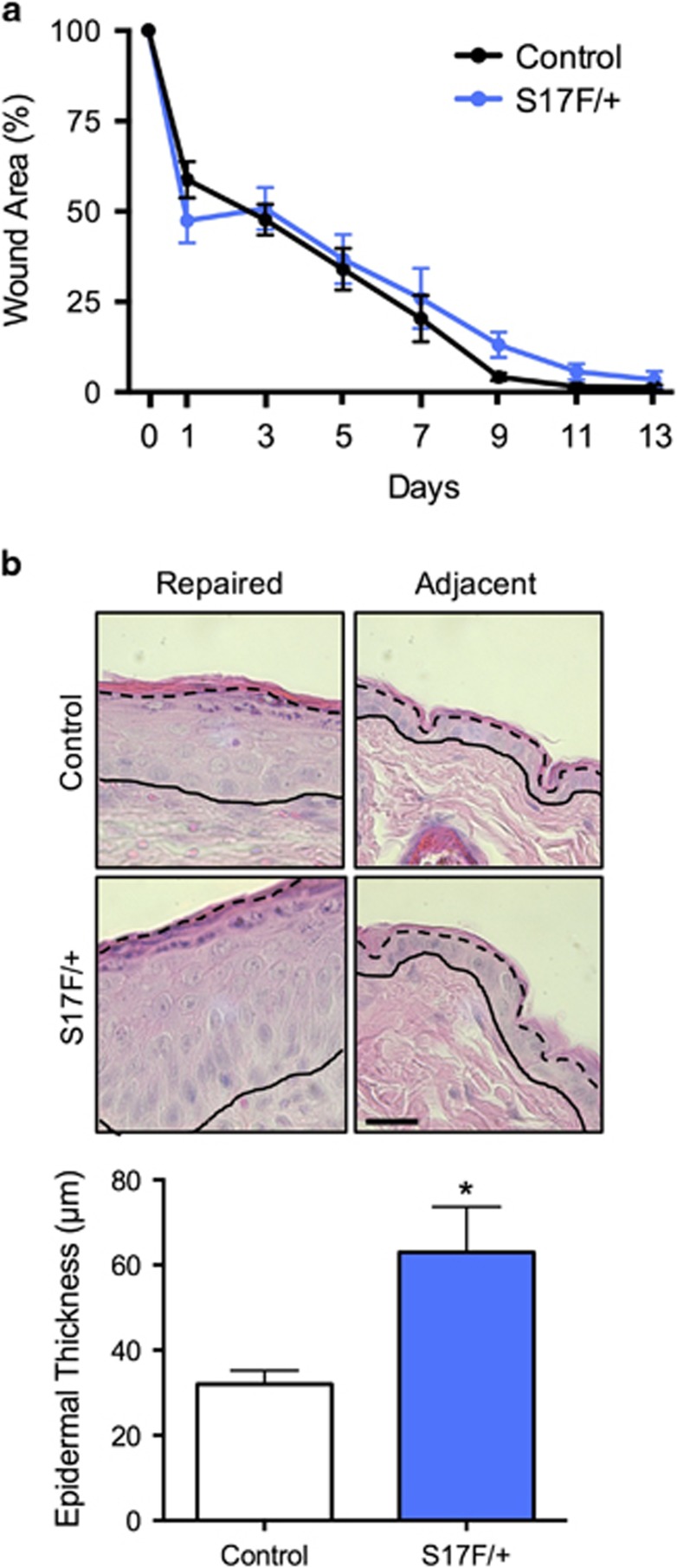
S17F/+ mice display normal wound closure, but exhibit abnormal epidermis remodeling. (**a**) Dorsal skin wound closure was found to be similar between S17F/+ mice and controls (c) (two-way ANOVA with repeated measures), however, epidermis remodeling following wound closure generated thicker epidermis in S17F/+ mice compared with controls (**b**) (unpaired *t*-test, *P<0.05, *N*=4). Scale bar, 10 *μ*m

**Figure 8:**
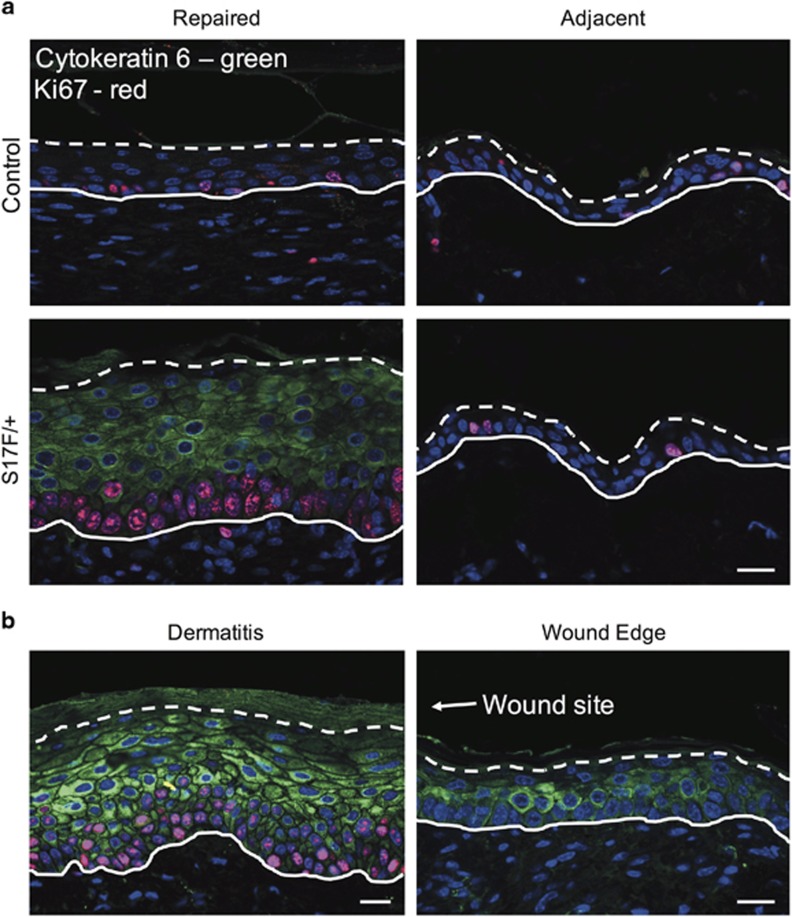
Repaired S17F/+ epidermis displays activated keratinocytes 14 days following wounding. (**a**) Repaired S17F/+ epidermis revealed prominent expression of cytokeratin 6 (green) and Ki67 (red) indicating activated keratinocytes similar to skin exhibiting dermatitis (**b**). Cytokeratin 6 expression diminishes at the wound edge (**b**). Scale bar in (**a** and **b**)=20 *μ*m

Since dynamic regulation of Cx26 coincides with different stages of wound healing, we tested whether S17F/+ mice displayed any overt wound healing defects. Between 3 and 4 months of age, a punch biopsy of depilated dorsal skin created a wound in which healing was monitored. S17F/+ mice displayed no differences in wound size at any stage of recovery (Figure 7a), however repaired epidermis at 14 days post-wounding was measurably thicker in S17F/+ mice suggesting possible aberrant epidermal remodeling in response to the wound (Figure 7b). Repaired epidermis also exhibited strong cytokeratin 6 and Ki67 expression (Figure 8a) similar to murine skin exhibiting dermatitis (Figure 8b). There were also no detectible differences in inflammatory cell invasion between control and S17F/+ repaired epidermis 14 days post-injury (Supplementary Figure 2).

## Discussion

Connexin-linked skin diseases encompass a diverse array of congenital skin abnormalities wherein patients express connexin mutants with defects in cellular communication.^27^ Mouse models harboring connexin modifications have demonstrated that the epidermis relies on complex GJIC to coordinate a balance of cell proliferation and differentiation for the maintenance of rapid physiological turnover and resiliency to injury.^16, 25, 28, 29, 30, 31, 32^ Of particular interest are Cx26 and Cx30 linked skin diseases due to the large number of distinct gene mutations that lead to syndromic diseases featuring variable skin disorders.^5^ Patients dominantly expressing the Cx26S17F mutant are diagnosed with KIDS and display severe hearing, ocular, and skin phenotypes.^24, 33^ Although mutant Cx26 undoubtedly underpins this disease, specific pathogenic mechanisms are poorly understood and the question of whether these patients have wound healing defects has not been explored. In this study, we generated a novel keratinocyte-specific Cx26^CK14-S17F/+^ mouse to address these questions. We found that S17F/+ mice developed severe PPK wherein foot pad epidermis exhibited increased Cx26 expression and irregular localization profiles of epidermal connexins. Furthermore, S17F/+ foot pad epidermis exhibited elevated filaggrin expression, but normal expression of cytokeratin 14 and displayed normal proliferation. Primary keratinocytes isolated from connexin mutant neonates revealed altered GJIC and migration abilities in culture. S17F/+ skin exhibited aberrant epidermal remodeling during wound healing but wounds healed at a similar rate to littermate control mice.

Cx26S17F was first linked to KIDS in 2002 (ref. 24) and is now understood to form non-functional gap junction channels or hemichannels but does form hyperactive heteromeric hemichannels with wild-type Cx26 and Cx43 in culture.^22^ Many syndromic Cx26 mutants fail to form functional channels,^7^ however studies investigating separate KIDS linked mutants reveal that hyperactive hemichannels may be a strong etiological factor in KIDS.^22, 23, 34^ A mutant mouse globally expressing Cx26S17F was found to replicate KIDS skin and hearing phenotypes making it a suitable model for KIDS.^25^ However, one drawback with this model was a sharp loss of mouse viability. To alleviate this concern and to directly assess the role of the Cx26S17F mutant in the epidermis, we generated a keratinocyte-specific mutant mouse driven by the cytokeratin 14 promoter. We showed here that indeed epidermal expression of Cx26S17F produced the severe PPK seen in KIDS, as well as a short, blunted tail reminiscent of autoamputated digits in patients with the Cx26-linked Vohwinkel Syndrome.^35^ Furthermore, Cx26^CK14-S17F/+^ mice in this study were generally healthy suggesting that expression of the S17F mutant in other tissues such as liver, kidney, brain, and gut may have contributed to reduced viability seen in mice with global expression of the S17F mutant. We also showed that Cx26^CK14-S17F/+^ mice had an intact epidermal barrier as evaluated by using the exact protocol from Schütz *et al.*^25^ This observation was surprising and suggested that Cx26S17F expression in embryonic tissues may interfere with the establishment of the epidermal barrier *in utero* and contribute to their poor prognosis. Cx26 is indeed expressed in the labyrinth layer of the fetal mouse placenta and interestingly, Cx26 null embryos die by 11 days post conception due to a placental defect.^36^ Direct empirical comparison of the development of global and conditional mutant mice may shed light on the nature of the barrier defect and loss of viability in global mutant mice, and could further point to developmental complications in patients who harbor KIDS mutations. Nevertheless, the Cx26^CK14-S17F/+^ mouse model allowed us to evaluate the influence of the Cx26S17F mutant on the epidermis alone, and whether this expression profile can disrupt wound healing which involves numerous unaffected cell types such as fibroblasts, leukocytes, and endothelial cells.

The nearly universal feature of PPK in connexin-linked skin diseases suggests that proper GJIC is crucial to maintain highly stratified volar epidermis.^3^ We found an imbalance of keratinocyte proliferation and differentiation in foot pad epidermis of S17F/+ mice indicated by increased filaggrin expression yet normal expression of Ki67 and PCNA. Since filaggrin condenses the keratin cytoskeleton in differentiated keratinocytes, we surprisingly observed that suprabasal keratinocytes from S17F/+ foot pad skin did not appear to adopt a squamous morphology like those in control epidermis. We also showed that Cx26, Cx30, and Cx43 were not confined to specific keratinocyte layers but rather were expressed in most keratinocytes, further suggesting deregulated differentiation. Calcium homeostasis in the epidermis is thought to have a large impact on keratinocyte differentiation,^10^ and since Ca^2+^ mobilizing molecules such as IP_3_ and ATP are permissible by gap junctions and hemichannels, the calcium profile in lesioned epidermis is an attractive, albeit difficult element to assess. Interestingly, Bosen and colleagues showed that unlike control littermates, global Cx26^S17F/+^ mice exhibited large amounts of Ca^2+^ in the stratum corneum, suggesting that Cx26S17F may disrupt the calcium profile in the epidermis.^12^ As some connexin hemichannels are known to release ATP in response to mechanical stimulation,^2, 37, 38^ it is plausible that mutant connexins in the mechanical environment of volar skin could stimulate aberrant purinergic signaling that disrupts Ca^2+^ homeostasis and finally differentiation.^38, 39^ Furthermore, lesions of flexural skin regions are also commonly observed in KIDS and Vohwinkel Syndrome patients^3, 21^ further supporting mechanically stimulated Cx26 mutants in skin pathogenesis. Nevertheless, investigations into the mechanical sensitivity of Cx26 mutants are warranted to shed light on this notion.

Keratinocytes isolated from conditional Cx26^CK14-S17F/+^ mice demonstrated that physiological levels of the Cx26S17F mutant led to reduced gap junctional coupling and a similar reduction in collective cell migration. Following wounding, undamaged keratinocytes at the wound edge initiate re-epithelialization by migrating under the coagulum.^40^ During this process, a transient reduction of cell surface Cx43 favors cell migration by reducing cell-cell adhesion through its binding partner zona occludens-1, a protein linked to junctional complexes.^41^ Our results suggest that Cx26, which is thought to bind an ubiquitin-ligase protein^42^ can also influence cell migration through direct intercellular communication. Despite being well documented, the transient upregulation of Cx26 in keratinocytes at the wound edge is poorly understood.^17, 43, 44, 45^ However, our results suggest this may generate a highly coupled keratinocyte network to optimize collective cell migration. To our surprise, S17F/+ keratinocyte cultures also appeared to form fewer and smaller gap junction plaques in light of extensive Cx26 gap junction formation in intact S17F/+ epidermis. However, reduced gap junction formation by Cx26S17F has been reported in HeLa cells,^22^ therefore the disparagement of connexin expression between native epidermis and *in vitro* models highlights the importance of using mutant mouse models for understanding connexin-linked pathologies.

Because Cx26 is dynamically regulated during wound healing^14^ and KIDS patients develop inflammatory skin lesions,^21^ it raises questions as to whether KIDS patients have abnormal wound healing. There are currently no reports of KIDS patients exhibiting wound healing defects; however, the protective care these patients require^21^ and the rare nature of this disease argues that a wound healing defect may be under reported. In one study linking Cx26 levels to wound healing, mouse models with persistent epidermal Cx26 expression developed inflammatory lesions and wounds which displayed improper remodeling^30^ suggesting that Cx26 may contribute to the inflammatory and remodeling phases of wound healing. However, the S17F/+ mice used in our study displayed no overt wound closure defects nor evidence of differences in inflammatory cell invasion. However, further examination of repaired S17F/+ epidermis revealed prominent cytokeratin 6 and Ki67 expression indicating activated keratinocytes and a possible delay in the transition to normal keratinocyte differentiation.^46^ Despite this, if our findings can be extrapolated to humans, it would suggest that KIDS patients might only display mild defects in cutaneous wound repair.

Lastly, we also demonstrated that hyperkeratotic foot pad epidermis in S17F/+ mice had elevated levels of Cx26, suggesting that while unchallenged skin was relatively normal, skin challenged by weight bearing or wound healing may provide the conditions for the Cx26S17F mutant to disrupt epidermal physiology. Although the exact nature of these conditions remains uncertain, our findings support the notion that Cx26S17F disrupts epidermal homeostasis by deregulating keratinocyte differentiation. Despite subtle differences between murine and human epidermis, our findings strongly support the generation and assessment of animal models that mirror connexin-linked diseases. Fortunately, five mouse models feature *GJB2* modifications have helped elucidate the pathogenesis of KIDS and Vohwinkel Syndrome,^25, 28, 47^ and have suggested a role for Cx26 in both epidermal barrier establishment and cutaneous wound healing.^30^ Our study offers further evidence for the influence of Cx26 during cutaneous wound healing and illustrates a link between Cx26 disorders and aberrant keratinocyte differentiation.

## Materials and Methods

### Genetically modified mice

The Animal Care Committee at Western University approved all animal experiments in accordance with guidelines from the Canadian Council for Animal Care. Cx26^CK14-S17F/+^ mice were generated in-house, on a mixed C57BL/6 and 129Sv background. Heterozygous floxed mice (Cx26^floxS17F/+^ - generated by Schütz *et al.* and provided by the Bonn group) were crossed with homozygous cytokeratin 14 Cre mice (*Gjb2*^*tm2.2Kwi/Cnrm*^, Jackson Labs) which were obtained from Dr. L. Dagnino. Therefore, S17F/+ mice would be expected to express Cx26S17F heterozygously in basal cells of the epidermis, as well as the dental epithelium and oral ectoderm.^48^ All animal experiments used wild-type littermates as controls (referred to as Control). Efforts to generate homozygous Cx26^CK14-S17F/S17F^ failed, as these mice were not viable. Roughly equal proportions of male and female mice were used for all experiments.

### Mouse genotyping

Briefly, ear notches at the time of weaning were incubated overnight at 58 °C in a proteinase K solution composed of 0.6 mg of proteinase K (Cat# 25530-049; Invitrogen, Carlsbad, CA, USA) dissolved in buffer containing 20 mM Tris Cl pH 8.3, 50 mM KCl, 2.5 mM MgCl_2_, and 0.5% Tween 20. The digested tissue solution was incubated for 10 min at 95 °C and DNA amplification was carried out by polymerase chain reaction (PCR). PCR mixtures included 10x PCR buffer (Cat# 1360566; Invitrogen), 50 mM MgCl (Cat# 11392196; Invitrogen), 10 *μ*m dNTPs (Cat# 10297-018; Invitrogen), and Platinum Taq polymerase (Cat# 10966-018; Invitrogen). Samples were run for 40 cycles with the annealing temperature set to 57 °C. PCR products were run on a 2% agarose gel including 200 *μ*g of ethidium bromide, and gels were visualized under ultra-violet light.

### Skeletal staining

Seven day old mice were euthanized, neatly eviscerated to reveal the skeleton, and the skeleton was stained according to a modified protocol from.^49^ Skeletons were dehydrated in 95% overnight and fixed in acetone for 24 h. Skeletons were stained for 4 days using a solution containing 0.015% alcian blue, 0.05% alizarin red, and 5% acetic acid in 70% ethanol as described in Wang *et al.*^50^ Finally, skeletons were cleared using 1% KOH in distilled water until the stained vertebrae and intervertebral disks were clearly visible. Two animals per genotype were used for skeletal staining.

### Micro-computed tomography

Micro-computed tomography (*μ*CT) scans of euthanized 3-month-old S17F/+ and control whole mouse bodies were obtained using the eXplore speCZT *μ*CT scanner (GE Healthcare Biosciences, Little Chalfont, UK) at Robarts Research Institute (London, ON, Canada) to assess the bone structure underlying the S17F/+ truncated tail or skeletal abnormalities in the digits. Further technical specifications and image processing techniques are outlined in Caskenette *et al.*^51^ Briefly, scans were acquired with a voxel size of 100 *μ*m^3^ and 3D composite isosurface images were constructed and manipulated using MicroView software (GE Healthcare Biosciences). One S17F/+ and one control mouse were used to create isosurface images.

### Epidermal barrier assay

Neonatal epidermal barrier function was assessed as outlined in Schutz *et al.*^25^ Briefly, P1 neonates were CO_2_ euthanized and washed in a series of increasing methanol solutions (diluted in PBS) then submerged in an aqueous 0.2% toluidine blue solution for 15 min. Pups were then washed 3 × in 90% ethanol and once in distilled water before examination. Areas of blue/purple staining indicated epidermal dye penetration from a defective water barrier. Intentional barrier disruption was performed by making a small incision to the skin, or by repeatedly treating the skin with an acetone soaked cotton swab as indicated in Tsai *et al.*^26^ Images were captured with an iPhone 5S.

### Tissue lysates and immunoblotting

Foot pad skin was dissected, frozen with liquid nitrogen, and pulverized with a cold mortar and pestle over dry ice. Lysates were generated by dissolving the pulverized tissue in 2x immunoprecipitation buffer (containing 1% Triton X-100, 150 mM NaCl, 10 mM Tris, 1 mM EDTA, 1 mM EGTA, 0.5% NP-40, phosphatase inhibitors (1.0 mg of NaF, 1.0 mg of Na_3_VO_4_), and a Complete Mini protease inhibitor tablet (Cat# 10570500 Roche, Basel, Switzerland). Lysates were stored at −80 °C until electrophoresis. Protein concentration was determined with a standard bicinchoninic acid assay (Cat# 23225, Pierce, Waltham, UK). Lysates were run on a 12% polyacrylamide gel using SDS-PAGE and protein was transferred to nitrocellulose membranes with the iBlot dry transfer system (7 min transfer). Membranes were blocked in 3% BSA in PBST and probed with 1:1000 mouse anti-Cx26 (Cat# 138100, Invitrogen), 1:1000 rabbit anti-Cx30 (Cat# 71-2200, Invitrogen), 1:5000 rabbit anti-Cx43 (Cat# C6219, Sigma, St. Louis, MI, USA), 1:5000 mouse anti-GAPDH (Cat# 2145925, Millipore, Billerica, MA), 1:1000 mouse anti-CK14 (Cat# MA5-11599, ThermoFisher, Waltham, MA, USA), 1:1000 rabbit anti-filaggrin (Cat# PRB-417P, Covance, Princeton, NJ), and 1:400 rabbit anti-proliferating cell nuclear antigen (PCNA) (Cat# FL-261:sc-7907, Santa Cruz, Dallas, TX) antibodies. Secondary antibodies included AlexaFluor680 (Cat# A21076, Life Technologies, Carlsbad, CA, USA) and IRDye800 (Cat# 24058, Rockland, Pottstown, Philadelphia, PE, USA) and membranes were visualized using the Odyssey infrared imaging system. An unpaired *t*-test was performed on the mean intensity (K counts) of biological replicates.

### Immunohistochemistry and immunofluorescence imaging

Foot pad skin was fixed with 10% neutral buffered formalin overnight at 4 °C, paraffin embedded, sectioned, and stained with hematoxylin and eosin for histological analysis according to Stewart *et al.*^52^ Epidermal thickness measurements were recorded from histological images using ImageJ. Repaired dorsal epidermis was dissected 14 days following wounding and processed as described above. Sections were deparaffinized in xylene, and rehydrated in decreasing concentrations of ethanol (100, 95, 90, and 80%) then in distilled water. Sections were heated at 95 °C in a 10 mM sodium citrate buffer with 0.05% Tween 20 (pH 6.0) for 20 min and allowed to cool at room temperature before washing with PBST and incubating in 3% peroxidase blocking solution for 10 min. Finally, sections were blocked for 30 min in 2% BSA+0.2% Triton X-100 and labeled with 1:200 mouse anti-cytokeratin 6 (Cat# ab93279), 1:400 rabbit anti-Ki67 (Cat# ab66155, Abcam, Cambridge, UK), 1:300 rat anti-CD4 (Cat# 41-9766-080, ThermoFisher), mouse anti-CD68 (Cat# MCA1957, BioRad, Hurcules, California), and rabbit anti-neutrophil elastase (Cat# ab68672, Abcam). The secondary antibodies used were 1:500 anti-rabbit Alexa488 (Cat# A11008, Life Technologies), 1:500 anti-rabbit Alexa555 (Cat# A21429, Life Technologies), and 1:300 anti-rat Alexa555 (Cat# A21434, Life Technologies).

Separate foot pad skin samples were also fixed, cryopreserved in 30% sucrose in PBS, embedded in 1% low melting point agarose containing 18% sucrose and 0.01% NaN_3_, flash frozen with liquid nitrogen, and cryosectioned for immunofluorescent imaging. Frozen sections were blocked in 2% BSA+0.2% Triton X-100 and labeled with 1:400 rabbit anti-Cx26 (Cat# 512800, Invitrogen), 1:400 rabbit anti-Cx30, 1:500 rabbit anti-Cx43, 1:200 mouse anti-cytokeratin 14, 1:200 rabbit anti-filaggrin, and 1:400 rabbit anti-Ki67. The secondary antibodies used were 1:500 anti-rabbit Alexa488, 1:500 anti-rabbit Alexa555, and 1:500 anti-mouse Alexa488 (Cat# A11017, Life Technologies). Sections were counter-stained with Hoechst 33342, mounted using Airvol, and imaged with a Zeiss LSM 800 confocal microscope equipped with ZenWorks software. Images were captured from at least 3 biological replicates with a 40 × water-immersion objective.

### Wound healing assay

Three-month-old mice were anesthetized with isoflurane, administered the analgesics ketoprofen (5 mg/kg body weight) and buprenorphine (15 mg/kg body weight) subcutaneously, then a small section of dorsal hair was shaved and depilated with Nair, and the skin was sterilized using the standard 3-stage preparation. Wounds were performed using a 5 mm human punch biopsy to remove the full thickness of the skin as described in Churko *et al.*^53^ Briefly, dorsal skin was held taut and a punch biopsy tool was twisted on the skin with light pressure until a full thickness circle of skin was removed. Skin tension was released and allowed to retract for ~30 s before imaging. Wound inspection and imaging was performed the day of surgery, and every second day until fully healed. Wound sizes were measured using ImageJ by an investigator blinded to the mouse genotypes. A two-way ANOVA with repeated measures was performed on the mean wound area of biological replicates.

### Cell lines

Rat epidermal keratinocytes (REKs) originally described in Baden and Kubilus^54^ were provided by Dr. V. Hascall and verified as keratinocytes in that they could stratify to form organotypic epidermis. Cells were grown in DMEM (Cat# 11965-092, Life Technologies) containing 4.5 g/l glucose supplemented with 10% fetal bovine serum (Cat# 12484-028, Invitrogen), 2 mM l-glutamine (Cat# 25030-081, Life Technologies), 100 units/ml penicillin, and 100 *μ*g/ml streptomycin (Invitrogen). Cells were incubated at 37 °C and 5% CO_2_.^55^

### Primary murine keratinocyte culture

Keratinocytes were isolated from neonatal mice (P2-P3) according to Churko *et al.*^16^ with minor procedural adjustments. Briefly, complete body skin was carefully dissected from neonates was rinsed in Ca^2+^ and Mg^2+^ free PBS (Cat# 14190-144, Invitrogen) containing 5 *μ*g/ml gentamycin (Cat# 15750-060, Invitrogen). Skins were floated dermis side down over 1.5 ml of 50 cU/ml dispase (Cat#354235, Corning) at 4 °C overnight with gentle rocking. The epidermis was gently separated from the dermis, minced with sterile scissors, and incubated in 0.25% trypsin-EDTA (Cat# 25200-056, Invitrogen) for ~10 min at 37 °C. The cell suspension was removed and placed into 1.5 ml of Keratinocyte Serum-Free Medium (K-SFM) (Cat# 37010-022, Invitrogen) supplemented with 1.4 mM CaCl_2_ and 1.5 ml of stock trypsin neutralizer solution (Cat# R-002-100, Invitrogen), and centrifuged. The pellet was resuspended in K-SFM supplemented with 0.05 mM CaCl_2_, poured through a 70 *μ*m cell strainer (Cat# 21008-952, Falcon, Waltham, MA, USA), and plated in dishes pre-coated with 50 *μ*g/ml collagen I (Cat# 354236, BD Biosciences, San Jose, CA, USA). The next day, cells were washed 2 times with Ca^2+^ and Mg^2+^ free PBS then incubated in K-SFM containing 1.4 mM CaCl_2_. Cells were used for experiments between 48–72 h following initial plating.

### Calcein-AM dye recovery

Approximately 1 × 10^6^ primary mouse keratinocytes or REKs were grown on 35 mm glass bottom dishes (Cat# D35-14-1-U, Matsunami, Bellingham, Washington) and pre-loaded for 10 min at 37 °C in an isotonic 0.3 mM glucose solution containing 2 *μ*l/ml calcein-AM (Cat# C3100MP, Molecular Probes, Eugene, Oregon) dissolved in DMSO (Cat# D2650, Sigma-Aldrich). Cells were washed in PBS, replenished with K-SFM, and photobleached to <20% of original fluorescence intensity with a 488 nm argon laser at 50% strength. Images were subsequently captured every 10 s for ~10 min with a LSM 800 Zeiss confocal microscope and a 40 × water-immersion lens. A minimum of three images series (in which multiple cells were photobleached) were performed for each experiment. Each image series was exported to ImageJ and fluorescence recovery of photobleached cells were measured using the Time Series Analyzer V3 plugin. Fluorescence recovery curves displayed summarized data and a two-tailed student’s *t*-test was performed on the mean area under the curve (AUC) of biological replicates.

### Scratch-wound assay

Primary mouse keratinocytes were seeded at 1 × 10^6^ cells per 35 mm gridded dish (Cat# 83.1800.001, Sarstedt, Nümbrecht, Germany). Culture dishes were pre-coated with collagen as previously described and following 48 h of incubation in K-SFM containing 1.4 mM CaCl_2_, cells were rinsed with Ca^2+^ and Mg^2+^ free PBS and scraped with a P1000 pipette tip. Cells were then replenished with K-SFM containing no supplements or CaCl_2_ and images of 10 identical sections per dish (0.5 mm^2^), were captured at 24 and 48 h following the scrape. For each image, the gap between keratinocyte fronts was measured in five similar regions and an unpaired *t*-test was performed on the calculated migration distances of biological replicates.

### Statistical analysis

Graph Pad Prism version 6 was used for all statistical analysis and statistical significance was noted when *P*<0.05. All student’s *t*-tests were two-tailed. All histogram values represent the mean+S.E.M.
