# Correction to: Disease-linked connexin26 S17F promotes volar skin abnormalities and mild wound healing defects in mice

**DOI:** 10.1038/s41419-018-0639-1

**Published:** 2018-05-24

**Authors:** Eric Press, Katanya C Alaga, Kevin Barr, Qing Shao, Felicitas Bosen, Klaus Willecke, Dale W Laird

**Affiliations:** 1 Department of Physiology and Pharmacology, University of Western Ontario, London, ON Canada; 2 Department of Anatomy and Cell Biology, University of Western Ontario, London, ON Canada; 3 LIMES (Life and Medical Sciences Institute), Molecular Genetics, University of Bonn, Bonn, Germany

*Cell Death and Disease* **8**, e2845; 10.1038/cddis.2017.234; Article published online: 1 June 2017

Since publication of this article, the authors identified an error in the nomenclature of the cytokeratin 14 Cre mice in the Methods section. On PDF page 10, line 6 the phrase

“…..were crossed with homozygous cytokeratin 14 Cre mice (Gjb2tm2.2Kwi/cnrm, Jackson Labs)……”

Should read:

“…. were crossed with homozygous cytokeratin 14 Cre mice (Tg(KRT14-Cre)1Amc/J, Jackson Labs) ……”

The authors apologise for any inconvenience caused.

