# Supplementary material for: Disease-linked connexin26 S17F promotes volar skin abnormalities and mild wound healing defects in mice
Source: Cell Death Dis. 2017 Jun 1;8(6):e2845–. doi: 10.1038/cddis.2017.234 (PMC5520893; doi:10.1038/cddis.2017.234)
Supplement: Supplementary Information [file cddis2017234x1.pdf]

Supplementary Information

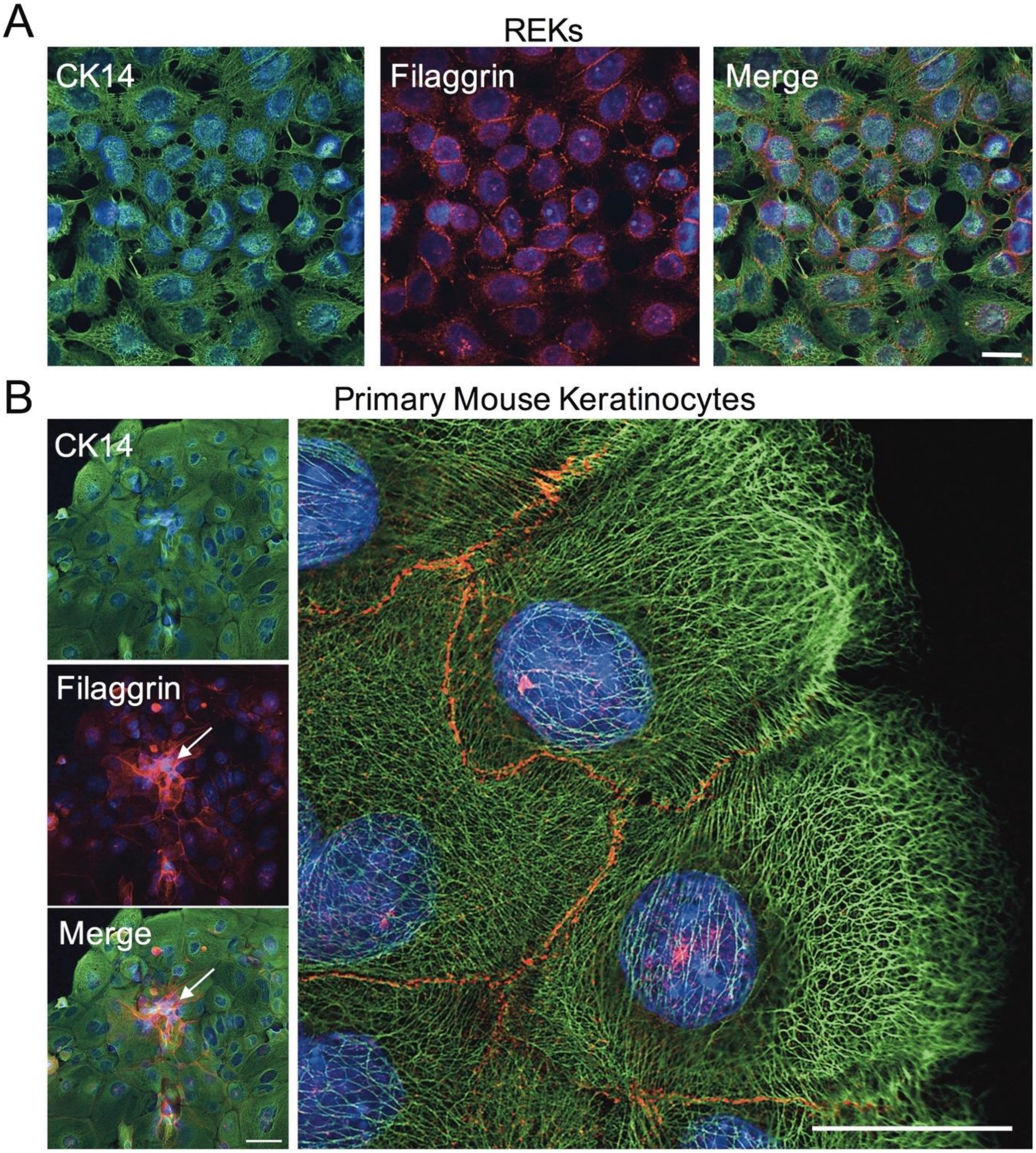

Fig. S1. Keratin filament expression in isolated keratinocyte cultures. Similar to well-characterized REKs (A), highly pure primary keratinocyte cultures (B) immunolabelled extensively with cytokeratin 14 (green) and demonstrated elevated filaggrin (red) expression in regions where cells began to stratify (arrows – left). Individual keratin filaments were visible under high magnification (right). Scale bar in (A) = 10 µm, (B-left) = 20 µm, (B-right) = 10 µm.

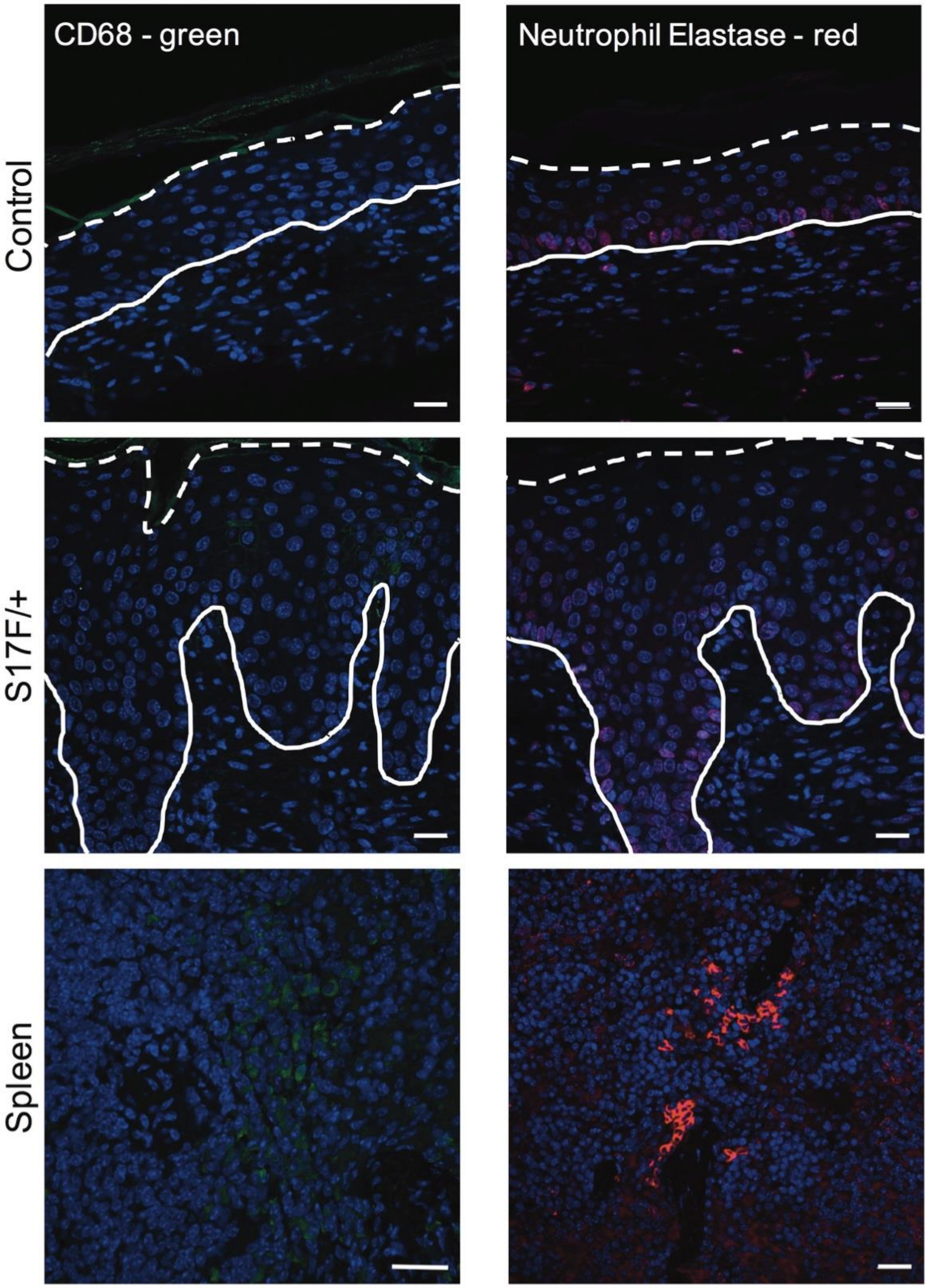

Fig. S2. Repaired epidermis does not exhibit evidence of immune cell invasion 14 days following wounding. Immunolabeling of control and S17F/+ dorsal skin revealed no invasion of macrophages (CD68), or neutrophils (neutrophil elastase). Mouse spleen tissue was used as a positive control wherein CD68 and neutrophil elastase positive cells can be observed. Complete and dashed lines indicate the dermis-epidermis boundary, and stratum granulosum-corneum boundary, respectively. Scale bar = 20µm.
